# UGT74AF3 enzymes specifically catalyze the glucosylation of 4-hydroxy-2,5-dimethylfuran-3(2H)-one, an important volatile compound in *Camellia sinensis*

**DOI:** 10.1038/s41438-020-0248-x

**Published:** 2020-03-01

**Authors:** Yongxian Chen, Xiangyang Guo, Ting Gao, Na Zhang, Xiaochun Wan, Wilfried Schwab, Chuankui Song

**Affiliations:** 1 State Key Laboratory of Tea Plant Biology and Utilization, International Joint Laboratory on Tea Chemistry and Health Effects, Anhui Agricultural University, 230036 Hefei, Anhui P. R. China; 2 Biotechnology of Natural Products, Technische Universität München, Liesel-Beckmann-Str. 1, 85354 Freising, Germany

**Keywords:** Enzymes, Metabolism

## Abstract

4-Hydroxy-2,5-dimethylfuran-3(2H)-one (HDMF) is an important odorant in some fruits, and is proposed to play a crucial role in the caramel-like notes of some teas. However, its biosynthesis and metabolism in tea plants are still unknown. Here, HDMF glucoside was unambiguously identified as a native metabolite in tea plants. A novel glucosyltransferase UGT74AF3a and its allelic protein UGT74AF3b specifically catalyzed the glucosylation of HDMF and the commercially important structural homologues 2 (or 5)-ethyl-4-hydroxy-5 (or 2)-methylfuran-3(2H)-one (EHMF) and 4-hydroxy-5-methylfuran-3(2H)-one (HMF) to their corresponding β-D-glucosides. Site-directed mutagenesis of UGT74AF3b to introduce a single A456V mutation resulted in improved HDMF and EHMF glucosylation activity and affected the sugar donor preference compared with that of the wild-type control enzyme. The accumulation of HDMF glucoside was consistent with the transcript levels of UGT74AF3 in different tea cultivars. In addition, transient *UGT74AF3a* overexpression in tobacco significantly increased the HDMF glucoside contents, and downregulation of *UGT74AF3* transcripts in tea leaves significantly reduced the concentration of HDMF glucoside compared with the levels in the controls. The identification of HDMF glucoside in the tea plant and the discovery of a novel-specific UDP-glucose:HDMF glucosyltransferase in tea plants provide the foundation for improvement of tea flavor and the biotechnological production of HDMF glucoside.

## Introduction

Plants produce a multitude of volatile compounds that play key roles in biological interactions^1,2^. Tea plants synthesize, accumulate, and emit many volatile compounds, which are characteristic metabolites and play an important role in the quality of tea. The formation of volatile constituents in various teas during the manufacturing process has been intensively studied, and ~700 volatiles have been identified, including esters, aldehydes, ketones, alcohols, terpenes, and furanones^3,4^. Among them, 4-hydroxy-2,5-dimethylfuran-3(2H)-one (HDMF) is considered an exceptional volatile because of its low-odor threshold in some fruits, attractive odor qualities, and remarkable odor-enhancement effects^5–8^. HDMF has a strong caramel-like aroma similar to that of its structural homologues 4-hydroxy-5-methyl-3(2H)-furanone (HMF, norfuraneol) and 2 (or 5)-ethyl-4-hydroxy-5 (or 2)-methyl-3(2H)-furanone (EHMF, homofuraneol). HDMF has been identified as a prominent volatile in pineapples^9^, strawberries^10^, grapes^11^, and tomatoes^12^, and it has also been found in black tea, where it contributes to the sweet and caramel-like odorants^4,13,14^.

Glycosylation is a major plant metabolite modification that plays an important role in many aspects, such as detoxification, transportation, and storage^15^. In addition, glycosylation also ensures the chemical stability and water solubility of plant metabolites while reducing chemical reactivity and toxicity^16^. Glycosylation also contributes to the storage of secondary metabolites and transport in plants^17^. Glycosylation is strictly controlled by glycosyltransferases (UGTs), which catalyze the transfer of activated sugar molecules to receptors to form corresponding glycosides. Although the study of glycosyltransferases is of great significance, there are few studies on glycosyltransferases, especially those related to volatiles^15^. In mature strawberry fruits, a large amount of HDMF was transformed into HDMF β-D-glucoside and accumulated^18,19^. The glucosylation of HDMF affects the development of plant volatiles, because HDMF glucoside is less volatile than HDMF and odorless^7,20^. Several *UGT* genes were isolated from strawberries whose encoded proteins catalyze the glucosylation of HDMF, including FaGT2^21^, UGT71K3^22^, and UGT85K16^23^. One glucosyltransferase gene, *UGT85K14*, was isolated from a grapevine cultivar^11^.

More than 300 UGTs were found in the tea plant genome^24,25^, but the function of most of them is unknown, except for that of four UGTs (UGT78A14, UGT78A15, UGT82A22, and UGT73A20), which exhibited catalytic activity toward phenolic acids and flavonoid^26,27^, and three UGTs involved in the glycosylation of geraniol^28^ and (Z)-3-hexen-1-ol^2^. However, the genes coding for HDMF glycosyltransferases in tea plants are not yet known.

In this study, HDMF glucoside was unambiguously identified as a native metabolite in tea plants by comparison of its retention time and accurate mass with those of an authentic standard. In addition, a novel glucosyltransferase UGT74AF3a and its allelic protein UGT74AF3b were identified to catalyze the glucosylation of HDMF and EHMF. The site-directed mutagenesis of UGT74AF3b led to the identification of one amino acid at the C-terminal end that influenced HDMF and EHMF glucosylation activity and sugar-donor preference compared with that of the wild-type control enzyme. In addition, the correlation between UGT74AF3 transcript level and HDMF glucoside accumulation in different tea cultivars was studied. The in planta function of UGT74AF3 was assessed by overexpression and silencing in tobacco and tea leaves, respectively. The identification of HDMF glucoside in the tea plant and the discovery of a novel-specific UDP-glucose:HDMF glucosyltransferase provides the foundation for improvement of tea flavor and the biotechnological production of HDMF glucoside.

## Materials and methods

### Plant material

Several cultivars of tea plants, including *Camellia sinensis* var. *sinensis “*Shuchazao”, “Yingshuang”, “Mingxuan213”, “Zhenghedabai”, “Huangqi”, “Mingshanbaihao”, and “Fudingdabai”, were collected from the Tea Plant Cultivar and Germplasm Resource Garden of Anhui Agricultural University (Guohe Town) and frozen in liquid nitrogen. All of the tea samples were stored at –80 °C until use.

### Chemicals and reagents

4-Hydroxy-2,5-dimethylfuran-3(2H)-one (HDMF), 2 (or 5)-ethyl-4-hydroxy-5 (or 2)-methylfuran-3(2H)-one (EHMF), 4-hydroxy-5-methylfuran-3(2H)-one (HMF), naringenin, eugenol, vanillic acid, salicylic acid, 1-naphthol, farnesol, sorbic acid, nerolidol, geraniol, fisetin, vanillin, chlorogenic acid, 2-naphthol, caffeic acid, methyl salicylate, ferulic acid, 1-octanol, and myricetin were purchased from Sigma-Aldrich (Shanghai, China). UDP-glucose, UDP-galactose, and UDP-glucuronic acid were purchased from Promega (Madison, USA). UDP-Xylose was purchased from Angfei Biotech Co., Ltd. (Guangzhou, China).

### RNA isolation, cDNA cloning, and sequence analysis

For analysis of gene expression, the leaves of *Camellia sinensis* var. *sinensis*. “Shuchazao” were collected for RNA extraction. The total RNA was isolated by RNAiso-mate for plant tissue (Takara, Dalian, China) and RNAiso Plus (Takara, Dalian, China). The cDNAs were amplified using Phusion® High-Fidelity DNA Polymerase (New England Biolabs, MA, USA), and the PCR products were purified via a MiniBEST agarose gel extraction kit (Takara, Dalian, China). The amplified PCR product was ligated into the pGEX-4T1 vector and transformed into TransT1-competent cells for sequencing.

### Quantitative real-time PCR analysis

Real-time PCR was performed according to our published protocols^2^ with gene-specific primers (Supplementary Table S1). qRT-PCR was carried out with the CFX96™ real-time system (Bio-Rad, USA). The two-step temperature program was 95 °C for 3 min, 40 cycles of 95 °C for 10 s, and 62 °C for 30 s in 96-well optical reaction plates. The glyceraldehyde-3-phosphate dehydrogenase (GAPDH) gene was used as an internal reference gene^2^, and the relative gene expression was calculated using the 2^–ΔΔCT^ method^29^.

### Heterologous protein expression and purification

The full-length sequence of *UGT74AF3* was digested with BamH1 and Smal1, and the resulting gene fragments were subcloned into the expression vector pGEX-4T-1. The recombinant plasmids were transformed into *E. coli* strain BL21 (DE3) pLysS cells. The empty expression vector pGEX-4T-1 was transformed into *E. coli* BL21 (DE3) pLysS cells, which served as the negative control. Protein expression was induced by adding IPTG (isopropyl-β-D-thio-galactopyranoside) at a final concentration of 1 mM when the OD_600_ of the culture was between 0.6 and 0.8. The cultures were incubated at 16 °C with oscillation at 150 rpm overnight. The next day, the proteins were purified using GST-binding resin (Novagen, Darmstadt, Germany) following the manufacturer’s protocol. The protein concentration was determined by the Bradford method, and bovine serum albumin served as the standard protein. The protein purity was further confirmed by SDS-PAGE.

### Enzymatic activity assay

The enzyme assays were carried out according to the methods described previously^2,21^. In the initial screening, the total volume of the enzyme reaction mixture (25 µL) contained 20 µL of Tris-HCl buffer (50 mM, pH 7.5, 10% glycerol, and 10 mM 2-mercaptoethanol), 1 µL of 2.5 mM UDP-glucose, 1 µL of 20 µM substrate solution, 1 µL of 50 mM DL-dithiothreitol, and 2 µL of purified protein (3 µg per reaction). The recombinant enzyme assay was carried out using the UDP-Glo^TM^ glycosyltransferase assay kit^30^. The optimum reaction temperature was determined to be in the range of 10–45 °C at pH 7.0. The optimum reaction pH was tested in the range from pH 3 to pH 10. Citrate, phosphate, and Tris-HCl buffers were used for the pH ranges 3–6, 6–8, and 8–10, respectively. To determine kinetic parameters, at least seven different substrate concentrations covering the range from 1 to 500 μM were used at the optimized conditions as previously described^2,21^.

### Identification of the products by LC–MS

The standard assays (200 µL) included 5 mM UDP-glucose, 200 µM substrate, and 10 to 20 µg purified protein. The enzyme assays were incubated at 30 °C for 3 h. The reaction was stopped and extracted twice with 200 mL of ethyl acetate. After vaporizing the organic solvent, the residue was further dissolved in 50 µL methanol/water (1:1, vol) for LC–MS analysis. LC–MS was performed with a reverse-phase C18 column (1.8 μm, 100 × 2.1 mm) at 40 °C. A DIONEX Ultimate 3000 UHPLC system (Thermo Fisher Scientific, Waltham, MA, USA) with an autosampler was utilized for all experiments. The solvent selection and LC parameters for product identification were set according to our published paper^2^. The products were identified by comparing their UV and MS spectra with those in the literature and reference material.

### Site-directed mutagenesis

A single A456V mutation of UGT74AF3b was conducted. The primers were designed based on sequence data of UGT74AF3b (Supplementary Table S3). PCR amplifications were carried out using the following conditions: 1 cycle of 2 min at 98 °C; 25 cycles at 98 °C for 10 s, 58 °C for 30 s, and 72 °C for 3 min; and a final extension at 72 °C for 5 min. The mutant gene was confirmed by sequencing. Mutant proteins were expressed and purified using the same procedure described above for native proteins.

### Construction and transformation of the overexpression plasmid

Full-length cDNA of *UGT74AF3a* from tea leaves was amplified by PCR using specific primers (Supplementary Table S2), and transferred to the expression vector pBI121. PBI121 with an intron containing the GUS gene was treated as the control. *Agrobacterium tumefaciens* strains AGL0 with pBI121-UGT74AF3a and pBI121 vectors were cultured at 28 °C in the LB medium containing appropriate antibiotics. When the bacteria reached an OD_600_ of 0.8, the cells were harvested and resuspended in modified MMA medium (MS salts, 10 mM MES pH 5.6, and 20 g L^−1^ sucrose). Transient experiments in tobacco leaves have been described previously^2^.

### Gene suppression of *UGT74AF3a* in tea using AsODNs

The previously described procedure for gene suppression in tea leaves was modified^31^. Candidate sequences of antisense oligonucleotides (AsODN) containing complementarity to the segment of the target gene were selected by using Soligo software^32^. AsODNs and special primers (Supplementary Table S4) were synthesized by Beijing Genomics Institute (BGI). To validate the suppression of *UGT74AF3a*, AsODN was used, and random nonsense ODNs were treated as the control. To determine the effects of *UGT74AF3a* gene suppression, naturally growing tender tea shoots containing two leaves were selected and put into Eppendorf tubes with 1 ml of a 20 μM AsODN-*UGT74AF3a* solution in this study.

### Metabolite analysis

HDMF glycosides were extracted and measured using the protocol described previously with slight modification^2^. For metabolite analysis, 50 mg of samples were extracted with 1 ml 75% (v/v) methanol twice. A chlorophenylalanine solution (3 μg mL^−1^) was added as an internal standard. The metabolites were sonicated for 20 min at 4 °C. After that, the mixture was centrifuged at 12,000 rpm and 4 °C for 10 min. The supernatants were used for HDMF glycoside analysis by LC–MS as mentioned above. For metabolite analysis, at least three independent biological replicates were examined.

### Phylogenetic tree and accession numbers

A phylogenetic tree was generated using a previously described method^2^. The following accession numbers were utilized: UGT85K14 (LC021362), UGT85K11 (ABB847092), UGT85K16 (LC312711), UGT76D1 (At2g26480), CsUGT78A14 (ALO19888.1), CsUGT78A15 (ALO1989.1), CsUGT84A22 (ALO19890), FaGT2 (AY663785), CsUGT73A20 (ALO19886.1), UGT94P1 (ABB847093), UGT71K3 (XP 004294260.1), UGT71C3 (At1g07260), UGT74F1 (At2g43840), UGT74F2 (At2g43820), UGT74D1 (At2g31750), and UGT74E2 (At1g05680).

## Results

### Identification of HDMF glucoside in tea plants

**Fig. 1:**
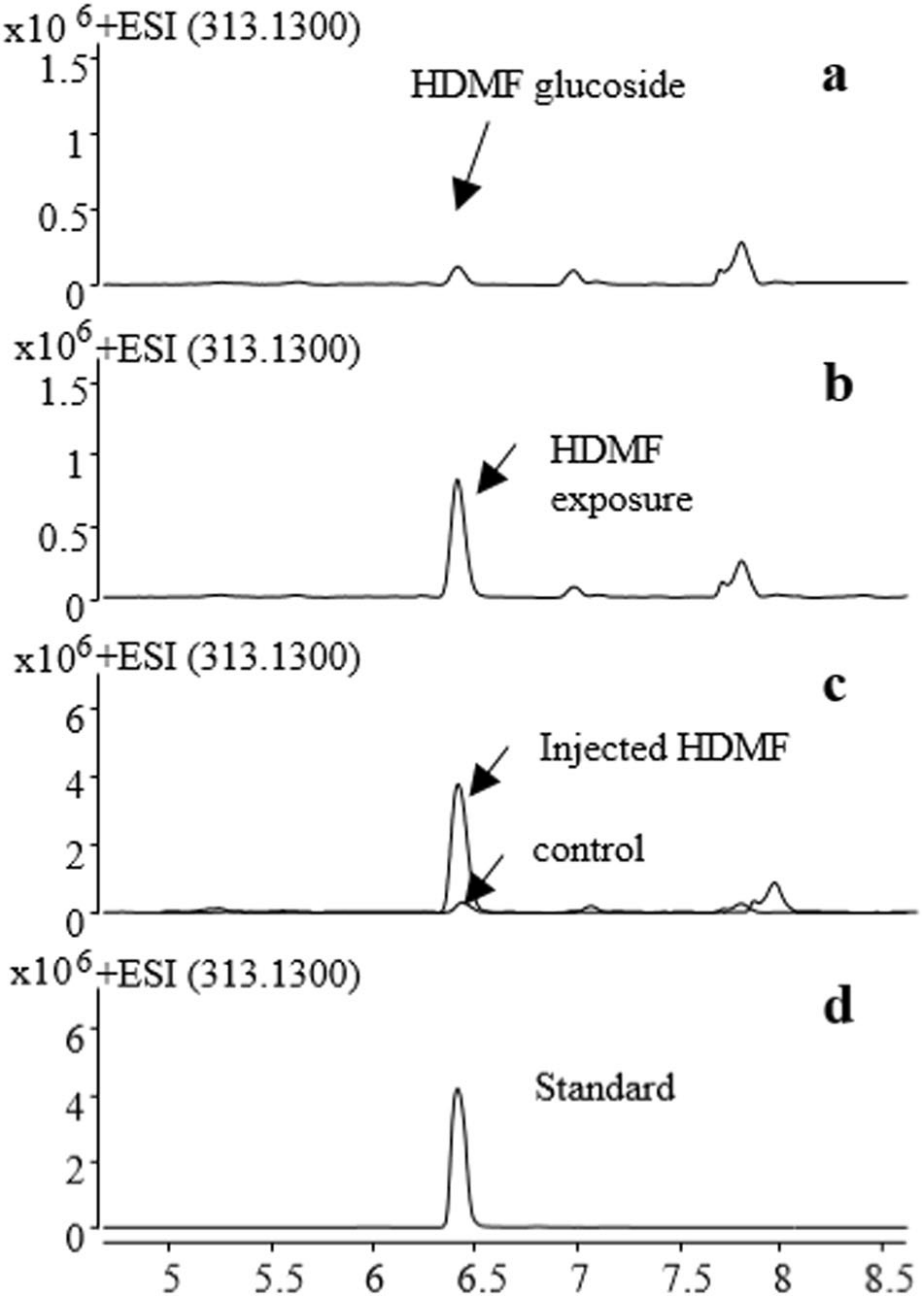
HDMF glucoside was identified in tea plants. The identity of HDMF glucoside in untreated leaves (**a**), tea leaves exposed to 20 mM HDMF for 24 h (**b**), and tea leaves injected with HDMF (**c**) was confirmed by LC–MS. DMSO was used as a control

To study whether HDMF glycosides were present in tea plants, the metabolites in young tissues of tea plants (*Camellia sinensis* var. *sinensis* “Shuchazao”) were extracted and analyzed by LC–MS. A peak with a retention time of 6.5 min was identified as HDMF glucoside by comparison of the retention time and accurate mass with those of an authentic standard (Fig. 1a, d). To prove that HDMF can be glucosylated in tea plants, undamaged tea leaves were exposed to 20 mM authentic HDMF in a closed glass vessel according to the method used in our previous research^2^. LC–MS analysis confirmed that the concentration of HDMF glucoside significantly increased twofold after exposure to HDMF for 24 h (Fig. 1b). Furthermore, when 20 mM authentic HDMF was directly injected into the tea plant, the concentration of HDMF glucoside rapidly increased to more than tenfold that of the control (Fig. 1c). All these data proved that HDMF glucoside is a native metabolite in tea plants; the tea plants could glucosylate HDMF, whereby HDMF glucoside was produced by an as-yet unknown UGT in the tea plants.

### UGT74AF3a and b specifically catalyze the glucosylation of HDMF

In *Arabidopsis*, 27 GTs, from ~107 enzymes, exhibited catalytic activity toward volatile compounds. The activities occurred in groups D, E, G, H, and L of the phylogenetic tree of the GT1 multigene family of *Arabidopsis*^26,33^. To study UGTs related to HDMF glycosylation, the candidate genes TEA012191, TEA016995, TEA031670, and TEA016084 were randomly selected from groups E, G, H, and L, respectively. Genes from group D were not selected because some genes in this group have been reported to encode flavonoid glucosyltransferases in tea plants^27^. TEA031670 could not be isolated as a full-length gene. The full-length sequences of TEA012191 and TEA016995 and two alleles of TEA016084 were obtained from the leaves of Shuchazao. The encoded UGTs were successfully expressed in *E. coli* with an N-terminal glutathione S-transferase (GST) tag.

**Fig. 2:**
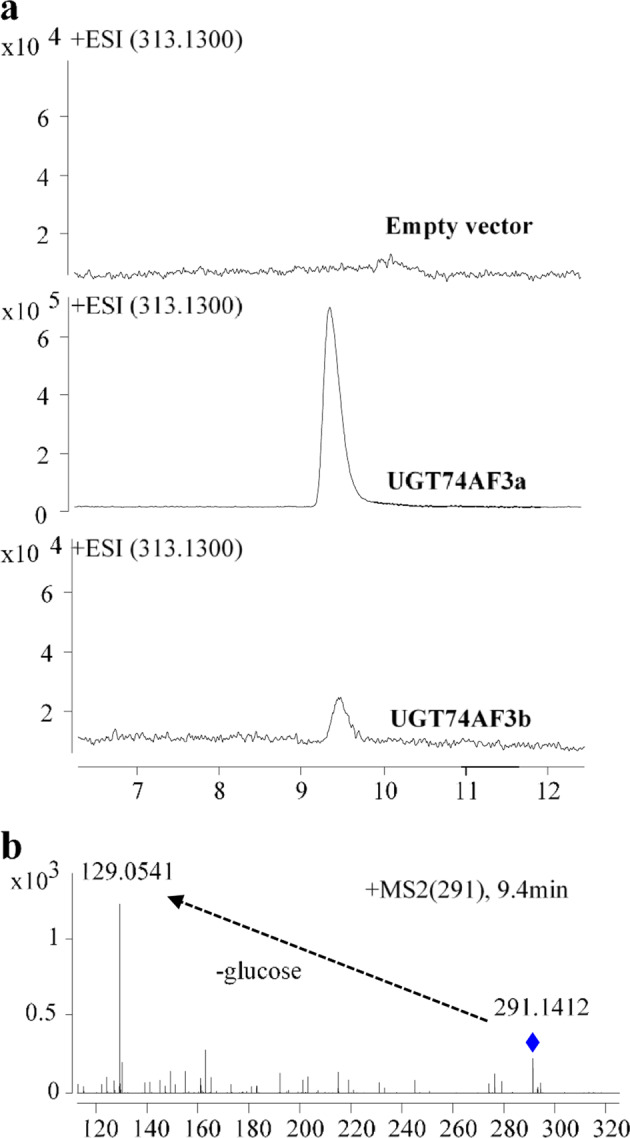
LC–MS/MS analyses of enzymatically formed products. LC–MS analysis of products formed by the control vector proteins UGT74AF3a and UGT74AF3b (**a**). Mass spectral analysis of the formed product (**b**)

**Fig. 3:**
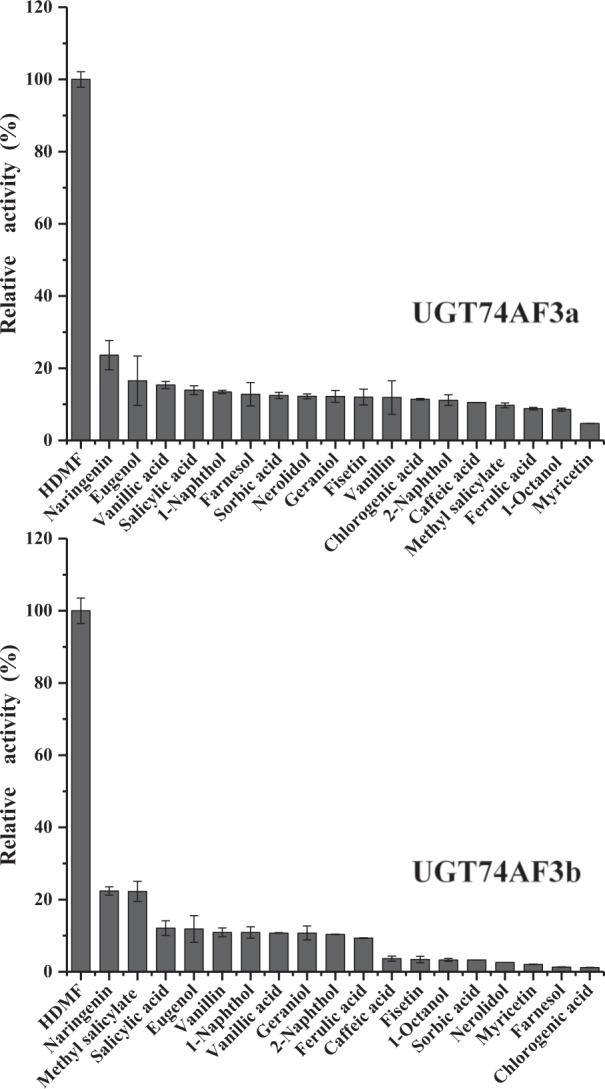
Activity screening of recombinant UGT74AF3a and b proteins with different substrates. The activity of HDMF was set as 100%. Values are expressed as the mean ± standard deviation of triplicate samples

The activities of recombinant proteins were first tested using the UDP-GLO glycosyltransferase assay with HDMF as the substrate. The recombinant protein encoded by TEA016084 catalyzed the formation of HDMF glucoside from HDMF and UDP-glucose, while the control vector proteins and the proteins encoded by TEA012191 and TEA016995 could not form HDMF glucoside. Two alleles of TEA016084 were isolated. The encoded proteins were subsequently assigned CsUGT74AF3a and b by the UGT Nomenclature Committee^34^ (Supplementary Fig. S2). The HDMF glucoside formed by both CsUGT74AF3a and b was identified by LC–MS (Fig. 2). The enzymatic activity of CsUGT74AF3a and b was further tested with 19 acceptor substrates (Fig. 3). The results indicated that the allelic UGT74AF3a and b proteins exhibited specific glucosylation activity toward HDMF (Figs. 2 and 3).

### UGT74AF3a and b exhibit different sugar-donor preferences

In addition to HDMF, its structural homologues (EHMF and HMF) were also used as substrates for UGT74AF3a and b, which exhibited the ability to use HDMF, EHMF, and HMF as substrates; however, UGT74AF3a exhibited a higher glucosylation activity toward HDMF, EHMF, and HMF than that of UGT74AF3b when UDP-glucose was used as a donor substrate (Supplementary Fig. S3).

Sugar-donor specificity was further analyzed. UGT74AF3a preferred UDP-Glc (100%) as a sugar donor over UDP-Gal (70–85%) and UDP-GA (70–80%) when HDMF and EHMF were used as donor substrates (Supplementary Fig. S3). However, when HMF was used as the substrate, the sugar preference was less obvious (Supplementary Fig. S3). Interestingly, UGT74AF3b also preferred UDP-Glc (38–42%) as a sugar donor, but the catalytic activities toward UDP-Gal and UDP-GA were very low (<20%) compared with that of UGT74AF3a when HDMF, EHMF, and HMF were used as substrates (Supplementary Fig. S3). Both UGT74AF3a and b could not use UDP- UDP-Xylose as a sugar donor when HDMF, EHMF, and HMF were used as substrates (Supplementary Fig. S3).

### Kinetic properties of the recombinant CsUGT74AF3a and b

The highest activity of both UGT74AF3a and UGT74AF3b was detected at pH 8.5 and 30 °C (Supplementary Fig. S1). The kinetic parameters of UGT74AF3a and b for HDMF were detected under the optimized conditions. The apparent *K*_M_ values of UGT74AF3a and b for HDMF were 1.59 and 5.14 µM, respectively (Table 1). The *k*_cat_/*K*_M_ ratios of UGT74AF3a and b for HDMF were 40.66 and 28.77 µM^−1^ s^−1^, respectively (Table 1).

**Table 1:** Kinetic data of recombinant UGT74AF3 for HDMF

Proteins	Substrate	*K*_M_ (µM)	Vmax (nKat/mg)	*K*cat/K_M_ (µM^−1^ s^−1^)
UGT74AF3a	HDMF	1.59 ± 0.19	4.23 ± 0.02	40.6
UGT74AF3b	HDMF	5.14 ± 1.58	11.38 ± 2.18	28.7

### Mutagenesis of UGT74AF3b at residue 456

**Fig. 4:**
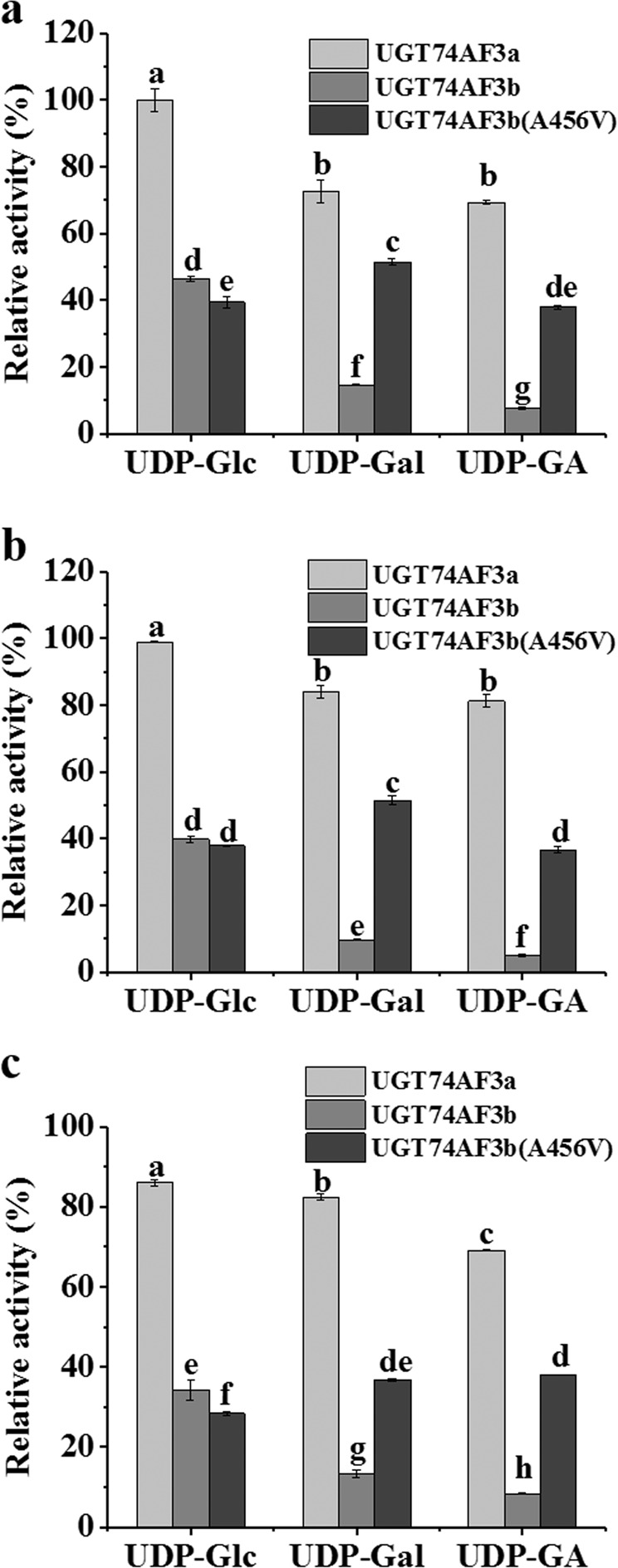
The relative enzymatic activities analysis of UGT74AF3 and A456V mutant. Comparison of the relative enzymatic activities of UGT74AF3a and b and the A456V mutant with HDMF (**a**), EHMF (**b**), and HMF (**c**) as substrates. Mean separation analysis of substrates was calculated using one-way ANOVA test and Duncan’s multiple-range test by SPSS 17.0. Means with different letters in each graph are significantly different (*P* < 0.05)

Although the similarity of the amino acid sequences of UGT74AF3a and UGT74AF3b was 99.43% (Supplementary Fig. S2), their activities toward HDMF and their sugar-donor preferences were significantly different. To investigate the role of amino acids in sugar-donor preference, an A456V mutant of UGT74AF3b was produced. The A456V mutant exhibited enhanced glucosylation activity toward HDMF, EHMF, and HMF in comparison with that of the wild-type UGT74AF3b enzyme when UDP-gal and UDP-GA were used as sugar donors (Fig. 4). In addition, the results also revealed that the A456V mutant protein exhibited a stronger preference toward UDP-gal than UDP-glc (Fig. 4). This result indicated that the residue at A456V played a key role in the sugar-donor preference of UGT74AF3.

### Transient overexpression of CsUGT74AF3a in tobacco

**Fig. 5:**
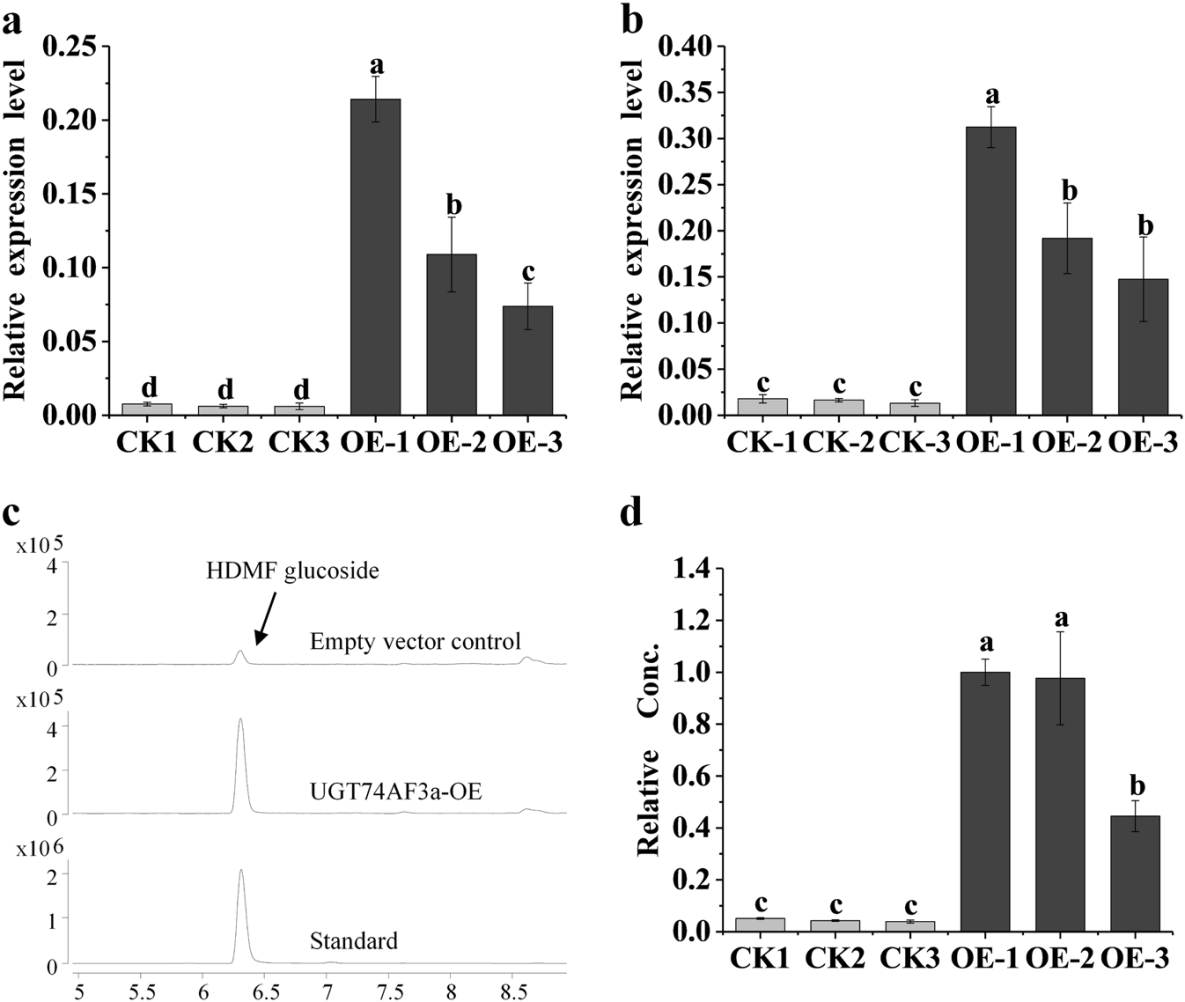
Overexpression of *UGT74AF3a* in tobacco. The relative mRNA levels of UGT74AF3a were measured by primer-1 (**a**) and primer-2 (**b**), and the HDMF glucoside product was analyzed by LC–MS (**c**, **d**). Duncan’s multiple-range test was carried out, and statistical significance was calculated with one-way ANOVA using SPSS 17.0 (*P* < 0.05). CK empty vector control, OE overexpression of UGT74AF3a-OE

To further investigate the role of UGT74AF3a in tobacco, transgenic tobacco studies were carried out as previously described^2^. Leaves were infiltrated using a suspension of *Agrobacterium tumefaciens* with empty pBI121-UGT74AF3a or pBI121 vectors, and were treated as the control. Relative mRNA levels of UGT74AF3a in *N. benthamiana* leaves were measured by two primers (Fig. 5a, b). The reaction products in both UGT74AF3a-overexpressing and pBI-intron control leaves were detected using LC–MS (Fig. 5d). The content of HDMF glucoside was markedly increased when *UGT74AF3a* was overexpressed (Fig. 5c), suggesting that HDMF can be formed in tobacco leaves, but the enzymes responsible for its glucosylation are not expressed. In addition, *UGT74AF3a* expression levels in individual tobacco leaves correlated very well with the concentration of HDMF glucoside (Fig. 5), showing that UGT74AF3 catalyzes HDMF glucoside production in tobacco.

### Functional assessment of UGT74AF3 in tea plants

**Fig. 6:**
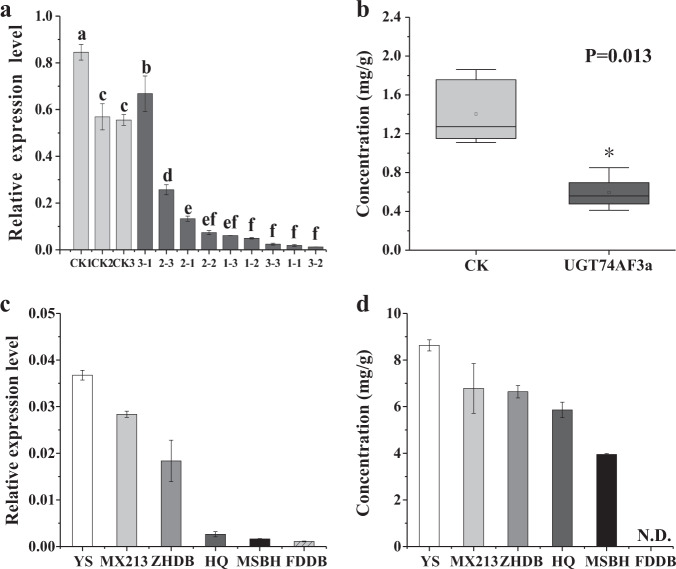
Functional characterization of UGT74AF3 in tea plants. The expression levels of UGT74AF3 (**a**, **c**) and the accumulation of HDMF glucoside (**b**, **d**) in tea leaves after treatment with AsODN-UGT74AF3 (a and b) and in different genotypes of the tea plant (**c**, **d**). Tea leaves treated with sense oligonucleotides were used as a control (CK). Three antisense oligodeoxynucleotides were designated as 1, 2, and 3. ND not detected. Statistical significance was calculated with one-way ANOVA using SPSS 17.0 (*P* < 0.05)

To determine the function of UGT74AF3 in HDMF catabolism in tea plants, the expression level of UGT74AF3 was transiently downregulated in *C. sinensis* leaves by gene-specific antisense oligodeoxynucleotide suppression according to Zhao et al.^31^. Three antisense oligodeoxynucleotides were designed (designated as 1, 2, and 3; Fig. 6). Tea plant leaves treated with sense oligodeoxynucleotides were used as a control. The expression level of *UGT74AF3* in tea leaves treated with AsODN_UGT74AF3 was significantly reduced compared with that in leaves of the control group, except for one sample treated with AsODN 3-1 (Fig. 6a). The content of HDMF glucoside in Cs*UGT74AF3*-silenced tea leaves was determined by LC–MS. The HDMF glucoside content was significantly reduced in the Cs*UGT74AF3*-silenced tea leaves compared with that in the control (Fig. 6b). The results indicated that UGT74AF3 can catalyze HDMF glucosylation in tea plants.

To further investigate the relationship between *UGT74AF3* gene expression and the concentration of glycosylation products of UGT74AF3a in different tea plant cultivars, several common tea cultivars were selected and analyzed. *UGT74AF3a* expression was detected in all cultivars, but the expression level of *UGT74AF3a* in Fudingdabai (FDDB) was the lowest (Fig. 6c). The concentration of HDMF glucoside was measured by LC–MS. The compound was detected in all cultivars, but not in FDDB (Fig. 6d). These results showed that there was a strong correlation between the mRNA levels of *UGT74AF3a* and the concentration of HDMF glucosides in different tea plant cultivars.

## Discussion

### Glucosylation of HDMF and phylogenetic analysis

HDMF is a key odorant in many fruits and was unambiguously identified as a native metabolite in tea plants in this study. In many aromatic plants, volatile compounds are stored as glycoside conjugates, which are water-soluble and odorless^15,28^. With regard to the biosynthesis of HDMF glucoside, some genes coding for HDMF UGTs have already been isolated and identified. The UGT85 family members UGT85K14 and UGT85K16 possess substrate specificity for HDMF compounds, and were isolated from the hybrid grapevine cultivar Muscat Bailey A (*Vitis labrusca* x *V. vinifera*) and strawberries (*Fragaria* *×* *ananassa*), respectively^11,23^. Although structurally distinct from the UGT85K family, UGT71K3 in strawberries could also catalyze the glucosylation of HDMF and EDMF (identical to EHMF)^22^, in addition to acylphloroglucinol, quercetin, 1-naphthol, and pelargonidin^22,35^. In tea plants, several UGTs have been cloned and functionally characterized, but only a few of them are involved in the glucosylation of volatiles. UGT85K11 from *Camellia sinensis* was recently reported to catalyze the formation of geranyl glucoside, and UGT94P1 specifically catalyzes the xylosylation of the 6′-hydroxy group of the sugar moiety of geranyl β-D-glucopyranoside and forms geranyl β-primeveroside^28^. CsUGT85A53 catalyzes the formation of (Z)-3-hexen-1-ol glucoside in tea plants and might be involved in plant defense and plant–plant interaction^2^. However, UGTs related to the glucosylation of HDMF in tea plants are still unknown. In this study, it was shown that UGT74AF3a and UGT74AF3b, which are members of the new UGT74AF subfamily, exhibit specific glucosylation activity toward HDMF, although they are structurally distinct from the UGT85K and UGT71 families. A previous study indicated that UGT85C2 and UGT74G1 could catalyze the glucosylation of steviol^36^. The apparent K_M_ values of UGT74AF3a (1.59 µM) and b (5.14 µM) for HDMF were similar to that of UGT85K16 (0.32 µM) from strawberries^23^, but significantly lower than that of UGT71k3a (900 µM) and UGT71K3b (406 µM) from strawberries^22^ and UGT85K14 (156 µM) from a hybrid grapevine cultivar^11^, suggesting that UGT74AF3a/b from tea plants showed a higher affinity for HDMF.

**Fig. 7:**
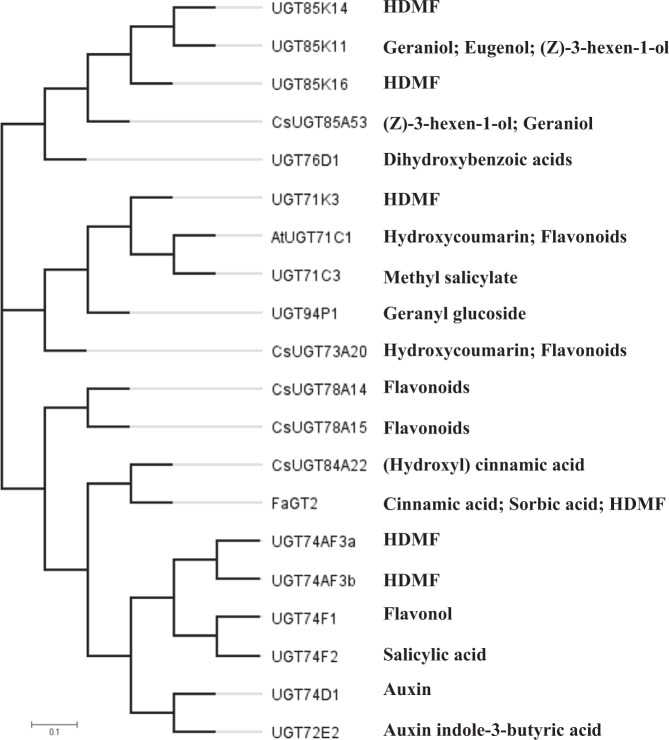
Phylogenetic tree of UGT74AF3a, UGT74AF3b and selected plant glycosyltransferases. The phylogenetic tree was generated by the neighbor-joining method within the MEGA 6.0 program using 1,000 bootstrap replications. The following accession numbers were utilized: UGT85K14 (LC021362), UGT85K11 (ABB847092), UGT85K16 (LC312711), UGT76D1 (At2g26480), CsUGT78A14 (ALO19888.1), CsUGT78A15 (ALO1989.1), CsUGT84A22 (ALO19890), FaGT2 (AY663785), CsUGT73A20 (ALO19886.1), UGT94P1 (ABB847093), UGT71K3 (XP 004294260.1), and UGT71C3 (At1g07260). The bar indicates a 0.1 amino acid substitution/site

Although phylogenetic analysis can predict the function of UGTs, some enzymes of the same branch in the phylogenetic tree may have different functions (Fig. 7). In our study, UGT74AF3 preferentially glucosylated HDMF (Fig. 2), while UGT74F1, UGT74F2, and UGT74D1 from *Arabidopsis thaliana* glucosylated flavonols, salicylic acid, and auxin, respectively^37–39^. In addition, a previous study indicated that UGT74E2 was involved in the modulation of the plant water-stress response^40^. Identification of other HDMF glucosyltransferases in a new family will provide new insight into the substrate specificity of UGTs and the molecular evolution of HDMF glucosyltransferases.

### Residue 456 determines sugar-donor preference of UGT74AF3

Diversity in nucleotide sequences of glycosyltransferases leads to diversity in their functions^41^. The structural information of glycosyltransferases plays an important role in studying the glycosyltransferase structure or mechanism. Although 3D structures of UGTs have already been reported^41–43^, the molecular mechanism of glycosyltransferase binding to substrates is still unknown. Previous studies indicated that the N-terminal amino acid residues of glycosyltransferase-encoded proteins are related to acceptor binding, while the C-terminal amino acid residues are closely related to that of donors^22,41,44–47^. In this study, site-directed mutagenesis at a single position (A456V) led to an increase in HDMF glucosylation activity of UGT74AF3b (Fig. 4), which confirmed that amino acids close to the C-terminus affect the enzymatic activity, while mutations in the N-terminus mainly modify the substrate preference^2,47^.

The comparison of two enzymes with almost identical primary sequences showed that the ability of the enzymes to differentiate between UDP-Glc and UDP-Gal as donor substrates was primarily due to a single residue, Glu303, in the C-terminus, which was not located in the active site^48^. In this study, the A456V mutant significantly increased the activity toward UDP-Glc and UDP-Gal when compared with that of wild-type UGT74AF3b when HDMF, EHMF, and HMF were used as substrates (Fig. 4). The discovery that residue 456 determines the sugar-donor preference of UGT74AF3 enhances our understanding of site-directed mutagenesis as an efficient method for improving and altering the sugar-donor preference of UGTs.

### In planta function assessment of UGT74AF3

For a long time, because the transgenic system of tea plants has not been successfully established, the in vivo functional verification of tea plant genes has only been carried out in heterologous plants^2,27^. In this study, the in planta function of UGT74AF3 was first investigated in tobacco, where overexpression of UGT74AF3a resulted in an increased HDMF glucoside content compared to that of the control (Fig. 5), suggesting that UGT74AF3 can glucosylate HDMF in tobacco.

**Fig. 8:**
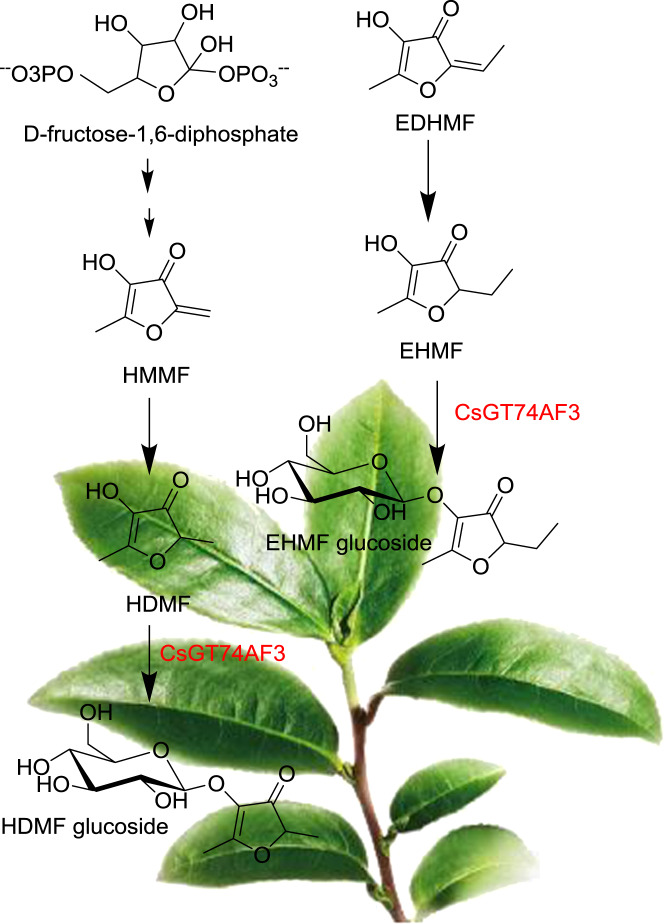
The proposed HDMF glucoside biosynthesis pathway in tea plants

Transient gene suppression using antisense oligonucleotides (AsODNs) is widely used in gene function analyses, and the use of AsODNs has been extended to various plant species^49^. A recent study showed that the functions of CsLIS/NES-1 and CsLIS/NES-2 in tea plants were successfully verified by AsODN. This method was further validated by treating tea seedlings with AsODN_PDS to suppress carotenoid production, suggesting that the AsODN method is effective in the tea plants^50^. To determine the function of UGT74AF3 in the tea plant, the expression level of *UGT74AF3* was transiently suppressed in *C. sinensis* leaves by gene-specific AsODN suppression according to Zhao et al.^31^. The expression level of *UGT74AF3* in tea leaves treated with AsODN_UGT74AF3 was significantly reduced in accordance with the HDMF glucoside content in comparison with that of the controls (Fig. 6). Finally, *UGT74AF3* gene expression was shown to correlate well with the concentration of HDMF glucosides in leaves of different tea plant cultivars (Fig. 6), suggesting that UGT74AF3 can catalyze HDMF glucosylation in tea plants (Fig. 8).

The identification of HDMF glucoside in tea plants and the discovery of a novel-specific UDP-glucose:HDMF glucosyltransferase provide not only the basis for the improvement of tea flavor, but also new insights for understanding the sugar-donor preference of UGTs and the molecular evolution of HDMF glucosyltransferases in plants.

## Supplementary information

Supplemental material
