# Supplementary material for: UGT74AF3 enzymes specifically catalyze the glucosylation of 4-hydroxy-2,5-dimethylfuran-3(2H)-one, an important volatile compound in Camellia sinensis
Source: Hortic Res. 2020 Mar 1;7:25. doi: 10.1038/s41438-020-0248-x (PMC7049299; doi:10.1038/s41438-020-0248-x)
Supplement: Supplementary file 1 — Supplemental material [file 41438_2020_248_MOESM1_ESM.pdf]

Table S1. Primers used for real-time PCR.

Name	Sequence (5' to 3')
UGT74AF3a-F1	TTGGGCTCTTGATGTTGCCA
UGT74AF3a-R1	ATGGCGTTTCACAGGACTCA
UGT74AF3a-F2	TGCCTTGGGCTCTTGATGTT
UGT74AF3a-R2	AACGATGGCGTTTCACAGGA

Table S2. Primers used for full-length amplification.

Name	Sequence (5' to 3')
UGT74AF3a-F	GGATCTGGTTCCGCGTGGATCCATGGAGACACCAAACAGAGC
UGT74AF3a-R	GCTCGAGTCGACCCGGGTTAGGATCGCACTAATTCAGCTAC
UGT74AF3b-F	GGATCTGGTTCCGCGTGGATCCATGGAGACACCAAACAGAGC
UGT74AF3b-R	GCTCGAGTCGACCCGGGTCATGCTAATTCAGCTACGAATTC

Table S3. Primers used for mutagenesis.

Name	Sequence (5' to 3')
UGT74AF3b-TF	GGATCTGGTTCCGCGTGGATCCATGGAGACACCAAACAGAGC
UGT74AF3b-TR	GCTCGAGTCGACCCGGGTTACACTAATTCAGCTACGAATTC

Table S4. Primers used for gene suppression.

Name	Sequence (5' to 3')
CK-AsON	CAAATGGAAGCTCAAGTGAA
Primer-1	TGGGTTGATGTGCCCTTGGG
Primer-2	TTGTCGTAGCCGCCATCGTC
Primer-3	GGCTTCGGGATTTTCGGCGT

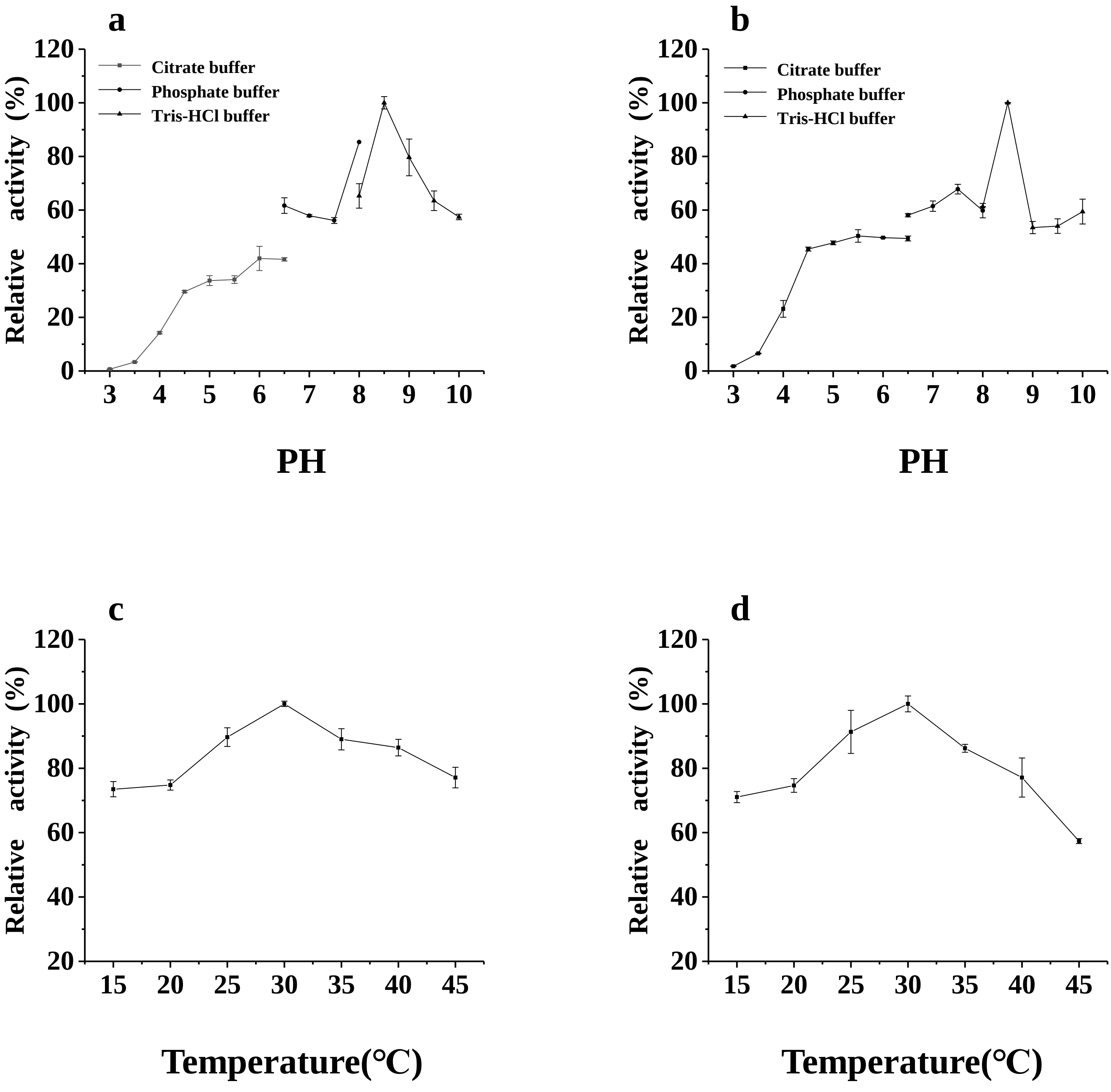

Fig. S1 The pH (a, b) and temperature (c, d) optima of UGT74AF3a (a, c) and b (b, d). Citrate buffer(pH 3 - 6.5), phosphate buffer (pH 6.5 - 8) and Tris-HCl buffer (8 - 10) were used. Values are means ± standard deviation of three replicates.

```
                    10        20        30        40        50        60        70
           ....|....|....|....|....|....|....|....|....|....|....|....|....|....|
UGT74AF3a  METPNRAYKAHVLVLPYPAQGHINPMLQFSKRLVARGVKATLANSVYISKSMHKDQIS-------TIDTD
UGT74AF3b  ..........................................................-------.....
UGT85K11   ---MGSRKQP.AVCV.F.........M.LA.L.HS..FYI.FV.TEFNHRRLLQSKGPEFLKGCADFQFE
UGT94P1    --MDSKKS.MN..M..WL.....T.F.ELA.K.TNKNFHIYFCSTPINLI.IK.RITDKYSLSIELVEIH

                    80        90       100       110       120       130       140
           ....|....|....|....|....|....|....|....|....|....|....|....|....|....|
UGT74AF3a  TFSDGHDDGGYDNAENPEAYLTKLRDVGSRTLASLIEKLNGLG--------RPVDALIYDGFLPWALDVA
UGT74AF3b  ...........................................--------...................
UGT85K11   SIP..LPPSDR.ATQD.PTLCIAM..NCLDPFRV.LK...NNNNSIASRQVPG.TCVVS..AMNF.MKA.
UGT94P1    LP.LPELPPH.HTTNGLPIH.NSTLKTAFEMASTSFSTILNTLS---------P.LV...VSPS..QST.

                   150       160       170       180       190       200       210
           ....|....|....|....|....|....|....|....|....|....|....|....|....|....|
UGT74AF3a  KELGILGVVFFTQTCA------------VNSIYYHVHEGLLSLPLSPDSTILLPGLPPLESCETPSFVYA
UGT74AF3b  ................------------..........................................
UGT85K11   E.A..PE.Q.W.ASACGFMGYLHYPQLVQRG.FPFKD.SFQ.DGSLDTTIDWI..MRNIRLKDM...IRT
UGT94P1    LSFD.PA.QLMITGAT---------------VVSFGQHMIKHCG---SVEFPF.AIKLQGFH..QFRHFV

                   220       230       240       250       260       270       280
           ....|....|....|....|....|....|....|....|....|....|....|....|....|....|
UGT74AF3a  YGLHPSFYDLLVNQFSNVDKADWVLFNTFYELEKEVVDWMS-KLWRVRTIGPTLPSMYLDQKLKDDIDYG
UGT74AF3b  .........................................-............................
UGT85K11   TDPNDILFNY.SEEVQ.CL..SAII....DT..HQ.LQAIAS.FHNIY....--L.LLSK.VIDGEFKSL
UGT94P1    ETVVKEYN.KQ.ASVNDQPSCNFM.Y...R...GKYI.YLP-------V..E------KKVVPVGPLVQ.

                   290       300       310       320       330       340       350
           ....|....|....|....|....|....|....|....|....|....|....|....|....|....|
UGT74AF3a  INLFKPHSTVCMNWLNAKPSSSVVYVSFGSMAQFEPEQMEEIAWGLNQSNYNFLWVVR------ATEEAK
UGT74AF3b  ..........................................................------......
UGT85K11   NSSLWKED.K.LQ..DT.EPN.....NY..ITVMTDQHLK.F....AN.KHP...I..P--DIVMGDS.I
UGT94P1    .DDDENEHSEIIQ..DN.GEY.TLF.....EYFMSK.EI....H..EL.MV..I....FPEVEKVEL.EA

                   360       370       380       390       400       410       420
           ....|....|....|....|....|....|....|....|....|....|....|....|....|....|
UGT74AF3a  LPNNFINDTAEKGLVVT-WSPQLEVLAHESIGCFVTHCGFNSVLEALSLGVPMVGVPYWSDQATNAKFVE
UGT74AF3b  .................-....................................................
UGT85K11   ..EH.VEE.KDR..L.S-.C..EQ..S.P...V.L....W..T..SICG...IICW.FFAE.Q..CRYAC
UGT94P1    ..KG..DRVG.R....EG.A..ARI.T.S.T.G..S..EW.....S.KF.....AI.MQYE.PL...L..

                   430       440       450       460       470       480       490
           ....|....|....|....|....|....|....|....|....|....|....|....|....|....|
UGT74AF3a  DVWGIGIRAKMDDKGIVRREVLEACMKEVFEGKKKNEVKMNAMKWKKLAKEALGDGGSSDKNIDEFVAEL
UGT74AF3b  ......................................................................
UGT85K11   TE....MEVNH.----.K.NEIV.LIN.ML..D.GKQMRKK.L.L..E.E..TDV..L.YN.F.RLIK.A
UGT94P1    E.G-VAAEVNR.IN.RLN..EIAQVIRK.VVE.SGEDIRIK.RIFGDKIRMKGDEEIDEAVEVLLQLCKD


           ....|..
UGT74AF3a  VRS----
UGT74AF3b  A------
UGT85K11   LHYCEQY
UGT94P1    .KLLKN-
```

Fig. S2 Comparison of the deduced amino acid sequences of UGT74AF3a / b, and two characterized volatiles related UGTs (UGT85K11 and UGT94P11) from ***Camellia sinensis*.**

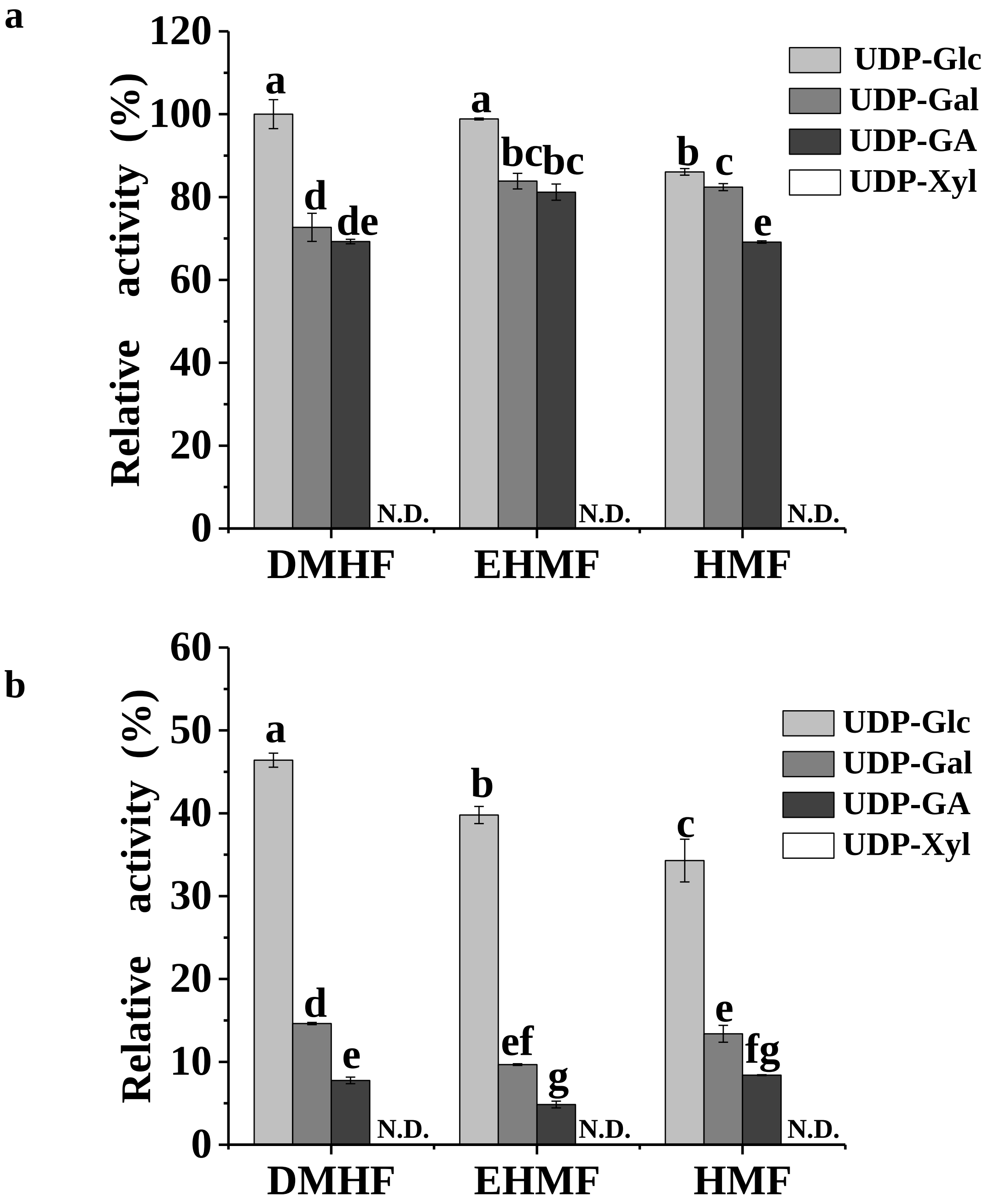

Fig. S3 Sugar donor preference of UGT74AF3a (a) and b (b) with DMHF and its structural analogs as acceptor substrates. The activity of DMHF with UDP-Glc was set as 100%. Values are means ± standard deviation of three replicates. UDP-Glc, UDP-glucose; UDP-Gal, UDP-galactose; UDP-GA, UDP-glucuronic acid.; UDP-Xyl,UDP-xylose; ND, not detected. Mean separation analysis of substrates was calculated using one-way ANOVA test and Duncan's multiple-range test by SPSS 17.0. Means by different letters in each graph are significantly different (P ＜0.05).
